# Mortality predictors in a cohort of patients with COVID-19 admitted to a large tertiary hospital in the city of São Paulo, Brazil: a retrospective study

**DOI:** 10.1590/1516-3180.2021.0914.R2.13062022

**Published:** 2022-09-12

**Authors:** Regina Maria Alexandre Fernandes de Oliveira, Milton Luiz Gorzoni, Ronaldo Fernandes Rosa

**Affiliations:** I Undergraduate Medicine Student, School of Medical Sciences, Santa Casa de São Paulo, São Paulo (SP), Brazil.; II MD, PhD. Associate Professor, Department of Internal Medicine, Irmandade da Santa Casa de Misericórdia de São Paulo (ISCMSP), São Paulo (SP), Brazil.; III MD, MSc. Instructor Professor, Department of Internal Medicine, Irmandade da Santa Casa de Misericórdia de São Paulo (ISCMSP), São Paulo (SP), Brazil.

**Keywords:** SARS-CoV-2, COVID-19, Mortality, Hospitalization, Prognosis, Predictors, Outcome, Parameters

## Abstract

**BACKGROUND:**

There is discrepant information across countries regarding the natural history of patients admitted to hospitals with coronavirus disease (COVID-19), in addition to a lack of data on the scenario in Brazil.

**OBJECTIVE:**

To determine the mortality predictors in COVID-19 patients admitted to a tertiary hospital in São Paulo, Brazil.

**DESIGN AND SETTING:**

A retrospective analysis of medical records of COVID-19 patients admitted to the Hospital Central da Irmandade da Santa Casa de Misericórdia of São Paulo.

**METHODS:**

Overall, 316 patients with laboratory-confirmed COVID-19 between March 1, 2020, and July 31, 2020, were included. The analysis included the baseline characteristics, clinical progression, and outcomes.

**RESULTS:**

The mortality rate of the sample was 51.27%. Age ≥ 60 years was determined as a risk factor after multivariate logistic regression analysis. Patients with an oxygen (O_2_) saturation ≤ 94% upon admission accounted for 87% of the deaths (P < 0.001). Vasoactive drugs were used in 92% (P < 0.001) of patients who progressed to death, and mechanical ventilation was employed in 88% (P < 0.001) of such patients. However, patients who received corticosteroids concomitantly with mechanical ventilation had a better prognosis than those who did not. The progressive degree of pulmonary involvement observed on chest computed tomography was correlated with a worse prognosis. The presence of thrombocytopenia has been considered as a risk factor for mortality.

**CONCLUSION:**

The main predictors of in-hospital mortality after logistic regression analysis were age, O_2_ saturation ≤ 94% upon admission, use of vasoactive drugs, and presence of thrombocytopenia.

## INTRODUCTION

Coronavirus disease (COVID-19) is a potentially fatal infection caused by the new severe acute respiratory syndrome coronavirus 2 (SARS-CoV-2) that was first reported in the city of Wuhan, China, in December 2019. Since then, the disease has achieved a pandemic status, assuming dramatic proportions, thus leading to a global public health emergency.^1,2,3^

In Brazil, the first case was officially confirmed on February 26, 2020, in the city of São Paulo, which has remained the epicenter of the country’s pandemic with the third highest total number of confirmed cases.^4,5^

Reports issued by the World Health Organization (WHO) on July 31, 2020, show that there is a significant difference in prognoses across countries, with a case fatality rate of the disease ranging between 3.53% (90,134/2,552,265 cases) in Brazil, 3.42% (150,054/4,388,566 cases) in the United States of America, and 14.21% (35,132/247,158 cases) in Italy. The reasons for such discrepancies remain to be fully elucidated, with population and genetic aspects, epidemiological control, population testing, and local management of the pandemic seeming to contribute to this situation.^6^

Moreover, in Brazil, approximately 70% of the population relied solely on the services of its National Healthcare System (known as the “Unified Health System,” or by its acronym in Portuguese: SUS, Sistema Único de Saúde),^7^ which rapidly led to hospital overcapacity and, therefore, possible preventable fatalities. In addition, hospitalization is known to have an array of negative impacts on the overall health of patients, such as secondary infections, needless procedures, psychological distress, and economic fallout of the health system that covers most of the nation’s population.

In this context, since most studies so far have focused either on a specific population subset, such as patients admitted to intensive care units or those with a specific disease, for instance or indiscriminately analyze data from public and private services altogether, there is still a gap in the literature on the mortality predictors in the general admitted population in a public hospital setting. Thus, this study aimed to characterize the predictive mortality factors in a cohort of patients admitted with COVID-19 to a tertiary public hospital in the city of São Paulo, Brazil, while also seeking to determine the clinical characteristics and prognostic factors of patients representing the most severe cases of the disease and hence requiring hospitalization.^8^

Therefore, these findings may support the creation and design of scientifically supported clinical management protocol for inpatient admission and hospitalized patient care in large public facilities.

## OBJECTIVE

This study aimed to determine the predictors of mortality in patients with COVID-19 admitted to a tertiary hospital in São Paulo, Brazil.

## METHODS

### Study design

This retrospective study was conducted at the Hospital Central da Irmandade da Santa Casa de Misericordia of São Paulo (ISCMSP), a large tertiary public hospital in São Paulo, Brazil. The Hospital Central has a total capacity of 559 beds, 550 of which are currently in use. At the time of this study, 152 of these beds were intended for COVID-19 patients, of which 72 beds were intended for intensive care.

Data were collected from the electronic medical records of patients with confirmed SARS-CoV-2 infection between March 1, 2020, and July 31, 2020.

The patients were selected from a list generated by the institution’s Nosocomial Infection Control Committee (CCIH, the Portuguese acronym for Comissão de Controle de Infecção Hospitalar), which included cases reported during the period of suspected COVID-19 cases due to the presence of respiratory symptoms, regardless of the primary cause of admission.

Of the 1840 selected patients, only 329 patients showed a positive result in the reverse transcription polymerase chain reaction (RT-PCR) assay, serology, or viral panel, performed either by Instituto Adolfo Lutz or the CientíficaLab, a laboratory associated with the institution, and were therefore considered for our analyses.

**Figure 1.**
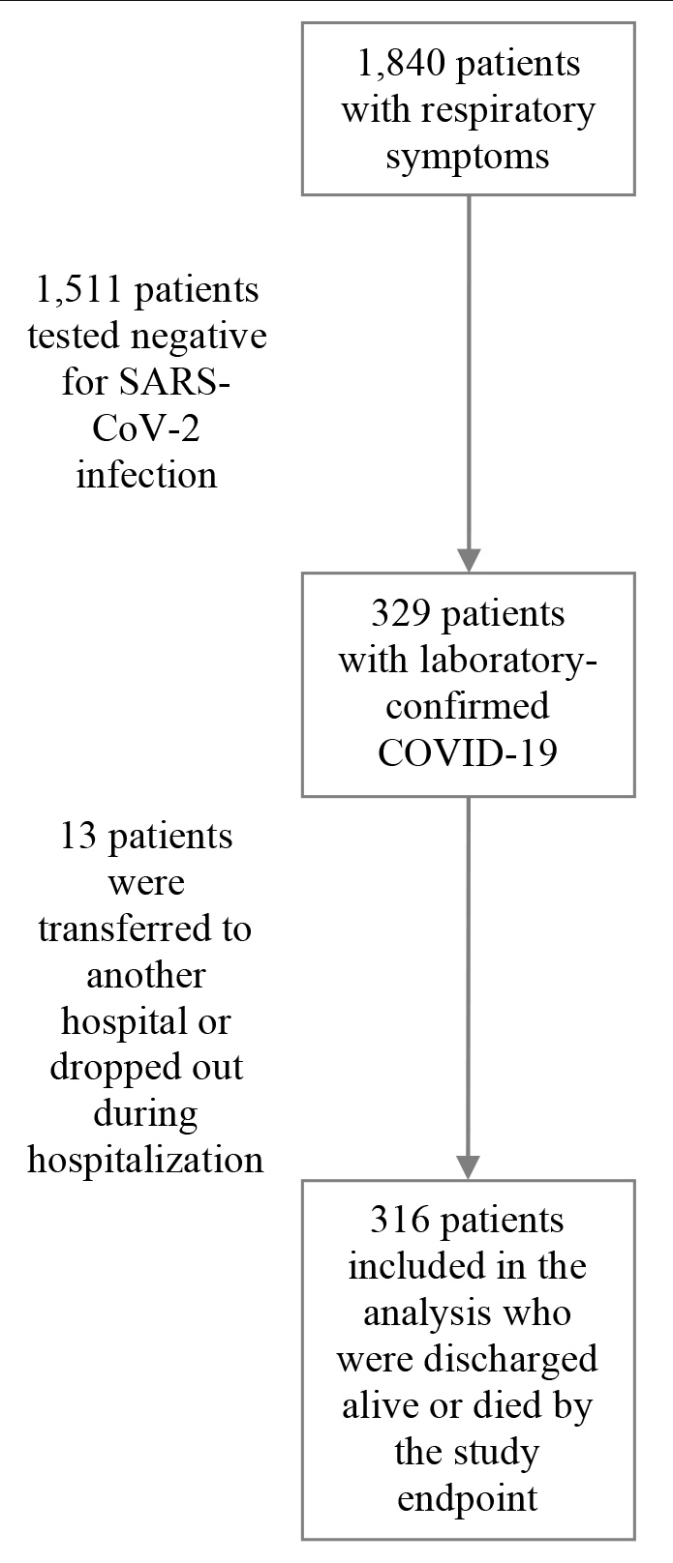
Flowchart for the selection of the study population.

An electronic form was developed to insert the data collected from the medical records (Sistema Soul MV, Versão SMA-PEP.2019.006. LTS [Recife, Brazil]). The following variables were collected:

- Demographic characteristics and information upon admission: age, sex, ethnicity, comorbidities, saturation (pulse oximetry), pulmonary involvement on chest computed tomography (CT), and indication for admission (due to COVID-19 or any other diagnosis).
- Progression and outcomes: use of vasoactive drugs, mechanical ventilation, hemodialysis, length of hospital stay, complications, and outcome (discharge from hospital or death). All patients who died during their hospital stay, regardless of the cause, were categorized under “death.” A total of 13 patients who were transferred or dropped out during hospitalization were excluded from this study. See Figure 1 for further details.

SARS-CoV-2 = severe acute respiratory syndrome coronavirus 2; COVID-19 = coronavirus disease.

Older adult patients were those aged over 60 years, as defined by the WHO for developing countries.^9^

This work was a part of project No. 4,893,387 (August 9, 2021) approved by the Research Ethics Committee (CEP, Portuguese acronym of Comitê de Ética em Pesquisa) at the institution where the study was carried out. Due to the retrospective nature of this study, the requirement for voluntary informed consent form was waived in accordance with the current regulations.

### Data collection

Comorbidities were reported on the basis of an initial anamnesis performed with the patient, their accompanying person, or as per the description contained in an electronic medical record from a previous time when the patient received health care services at the institution. The following were considered for analysis: chronic cardiovascular disease, systemic arterial hypertension, chronic kidney disease, use of corticosteroids, transplant recipients, chronic liver disease, chronic neurological disease, chronic neuromuscular disease, chronic lung disease, asthma, tuberculosis, smoking, diabetes mellitus, overweight, obesity, dementia, primary or secondary immunodeficiency, neoplastic disease, chronic hematologic disease, drug use, and pregnant or postpartum women.^10^

The vital signs considered were those upon admission to the COVID-19 department, either at the time of initial care in the emergency room or upon suspicion of nosocomial infection in patients who had previously been admitted to the hospital. A heart rate between 50 and 100 beats per minute was considered as normal,^11^ as was a respiratory rate between 12 and 20 breaths per minute.

The patients were considered to have been subjected to hemodialysis in all cases where this procedure had been employed during their hospital stay, whether due to a prior need or not.

The CT scans included in the analysis corresponded to the first study carried out within the first 24 h of patient admission to the COVID-19 sector.

With regard to the laboratory tests, normal values, such as those used by the clinicians at the laboratory where the tests were performed, were used as the parameters. Thus, in relation to the arterial blood gas test, an acid-base disorder was considered when the pH was below 7.35 or above 7.45, whereas hypoxemia was considered when the partial pressure of oxygen (PaO_2_) was lesser than 80 mmHg. With respect to the blood count, anemia was considered when the hemoglobin level was less than 12 g/dL, leukocytosis when the leukocyte count was greater than 10,000 cells/mm^3^, and thrombocytopenia when the platelet count was lesser than 140,000 platelets/mm³. In addition, prothrombin time (PT) was classified as normal between 11 and 12.5 s, normal activated partial thromboplastin time (aPTT) between 24 and 40 s, and altered D-dimer when values were above 0.50 μd/mL.

The use of any given medication was considered as positive if at least one dose thereof was administered.

### Statistical analysis

Data analyses were performed using the SPSS version 25.0 software (IBM Corporation, Armonk, New York, United States).

The statistical analysis of the study population was based on whether a given variable could be characterized as a mortality predictor.

Thus, several variables related to the clinical characteristics, supplementary tests, and treatments were compared according to patient progression (discharge from hospital or death). The corresponding frequencies were established, and Pearson’s chi-square test was used for the analysis, with a level of significance (P value) of 0.05.

Subsequently, to assess the risk factors associated with death, a multivariate logistic regression analysis was performed using the stepwise method, with a P value ≤ 0.05 requirement for data entry.

However, since the number of variables was quite extensive, pre-selection was carried out using bivariate analysis and setting a P value ≤ 0.20, according to Pearson’s chi-square test or Fisher’s exact test, along with excluding those with a data loss greater than 10%.

## RESULTS

A total of 316 patients admitted to the hospital with COVID-19 were included in the analysis. The mortality rate in this population was 51.27% (162/316; 95% confidence interval [CI], 45.75–56.78%).

In addition to the overall mortality rate of the study sample, three other groups were considered (clinical, supplementary, and treatment characteristics) to better elucidate the potential predictors of mortality.

However, it is noteworthy that for each item, the number of patients may vary according to the availability of data.

## Clinical characteristics that are mortality predictors

Of the 316 patients, 262 (82.91%) patients were community-acquired, and SARS-CoV-2 infection was the primary cause of hospitalization. The other 54 (17.09%) cases were nosocomial cases.

The average length of stay for the sample was 17.47 days (95% CI 15.13; 19.81), and 174 (55.06%) patients required admission to the intensive care unit during clinical progression, whereas 142 (44.94%) cases remained in the ward during the entire hospital stay.

In **eTable 1** in the Supplement (https://drive.google.com/file/d/1WwAiHPfkpZjC1wvaKArI6FpAoEtR-8e9/view), the data considered valid from 316 patients in the study population were evaluated for mortality predictors according to the hospitalization characteristics.

The mortality predictors were analyzed according to the clinical characteristics. Table 1 shows the main clinical characteristics of the sample. Most patients were observed to be Caucasian older adult men with comorbidities.

**Table 1.** General clinical characteristics of the study population

Clinical characteristics	Total	Discharge from hospital	Death	P
**Age (in years)**	316	154	162	< 0.001^*^
≥ 60	170 (54%)	64 (42%)	106 (65%)	
< 60	146 (46%)	90 (58%)	56 (35%)	
**Comorbidity**	309	153	156	< 0.001^*^
Yes	254 (82%)	112 (73%)	142 (91%)	
No	55 (18%)	41 (27%)	14 (9%)	
**Sex**	316	154	162	0.163 NS
Male	197 (62%)	90 (58%)	107 (66%)	
Female	119 (38%)	64 (42%)	55 (34%)	
**Ethnicity**	290	140	150	0.347 NS
Caucasian	191 (66%)	96 (69%)	95 (63%)	
Non-Caucasian	99 (34%)	44 (31%)	55 (37%)	

* Statistically significant; NS = not significant.

Subsequently, the same patient samples were assessed for vital signs upon admission. Oxygen saturation, as measured by pulse oximetry, had a significance probability of < 0.001; thus, it was statistically significant. Considering the admission data, patients with saturation ≤ 94% appeared to have a worse prognosis (Table 2).

**Table 2.** Vital signs upon admission

Vital signs upon admission	Total	Discharge from hospital	Death	P
**Sat (%)**	303	152	151	< 0.001*
≤ 94	240 (79%)	108 (71%)	132 (87%)	
> 94	63 (21%)	44 (29%)	19 (13%)	
**Heart rate (bpm)**	285	147	138	0.024 NS
Tachycardia	101 (35%)	60 (41%)	41 (30%)	
Normocardia	180 (63%)	87 (59%)	93 (67%)	
Bradycardia	4 (1%)	0 (0%)	4 (3%)	
**Respiratory rate (ipm)**	281	145	136	0.143 NS
Eupneic	101 (36%)	58 (40%)	43 (32%)	
Tachypneic	180 (64%)	87 (60%)	93 (68%)	

Sat = saturation; bpm = beats per minute; ipm = incursions per minute; NS = not significant; * statistically significant.

The use of vasoactive drugs, mechanical ventilation, and hemodialysis as potential prognostic indicators was also analyzed, with results shown in Table 3. However, after a multivariate logistic regression analysis, only the use of vasoactive drugs could be considered as a predictor of mortality.

**Table 3.** Hemodynamic progression of the study population

Hemodynamic progression	Total (n = 316)	Discharge from hospital (n = 154)	Death (n = 162)	P
**Use of vasoactive drug**				< 0.001^*^
Yes	171 (54%)	22 (14%)	149 (92%)	
No	145 (46%)	132 (86%)	13 (8%)	
**Mechanical ventilation**				< 0.001^*^
Yes	166 (53%)	24 (16%)	142 (88%)	
No	150 (47%)	130 (84%)	20 (12%)	
**Hemodialysis**				< 0.001^*^
Yes	62 (20%)	13 (8%)	49 (30%)	
No	254 (80%)	141 (92%)	113 (70%)	

* Statistically significant.

In **eTable 2** in the Supplement (https://drive.google.com/file/d/1WwAiHPfkpZjC1wvaKArI6FpAoEtR-8e9/view), symptoms are displayed according to their order of relevance as potential predictors. For this analysis, a report of a subgroup of 307 patients was considered, whereas those from which patient histories could not be retrieved were excluded. Of the symptoms listed, only the absence of fever was higher in the in-hospital mortality group.

### Supplementary characteristics serving as mortality predictors

To assess the possible associations between supplementary tests and hospitalization outcomes, subgroups were considered according to the available tests. Altogether, a chest CT analysis was performed for 116 patients (detailed in **eTable 3** in the Supplement: https://drive.google.com/file/d/1WwAiHPfkpZjC1wvaKArI6FpAoEtR-8e9/view), who represented the sample with such tests accompanied by their respective reports. In addition, entry oxygen saturation and the degree of impairment, as seen on chest CT, were correlated in a subgroup of 113 patients.

For chest CT analysis, pulmonary involvement was quantified in the imaging study, and the main findings were ground-glass opacity, consolidation, pleural thickening, and reticular opacities, with ground-glass opacity being the most common finding.

Three degrees of pulmonary involvement were considered (< 25%, between 25% and 50%, and > 50%); the greater the alterations, the greater the correlation with death, and therefore, the worse the prognosis. Additionally, when correlated with oxygen saturation upon admission lower or equal to 94%, the progressive severity of the pulmonary imaging findings remained associated with a poorer prognosis; taken together, these two constitute an important set of predictive mortality factors. However, this variable could not be included in the multivariate logistic regression analysis because of excessive data loss.

The laboratory characteristics were also evaluated, as shown in Table 4.

**Table 4.** Laboratory test results of the study population

Arterial blood gas test	Total (n = 258)	Discharge from hospital (n = 119)	Death (n = 139)	P
**Acid-base disorder**				0.320 NS
Yes	158 (61%)	69 (58%)	89 (64%)	
No	100 (39%)	50 (42%)	50 (36%)	
**Hypoxemia: PaO**_**2**_ **< 80 mmHg**				0.947 NS
Yes	161 (62%)	74 (62%)	87 (63%)	
No	97 (38%)	45 (38%)	52 (37%)	
**Blood count**	**Total (n = 292)**	**Discharge from hospital (n = 142)**	**Death (n = 150)**	**P**
**Anemia**				0.004*
Yes	149 (51%)	60 (42%)	89 (59%)	
No	143 (49%)	82 (58%)	61 (41%)	
**Leukocytosis**				< 0.001*
Yes	89 (30%)	29 (20%)	60 (40%)	
No	203 (70%)	113 (80%)	90 (60%)	
**Thrombocytopenia**				0.046*
Yes	24 (8%)	7 (5%)	17 (11%)	
No	268 (92%)	135 (95%)	133 (89%)	
**Altered PT and/or aPTT**	**Total (n = 254)**	**Discharge from hospital (n = 120)**	**Death (n = 134)**	**P < 0.001**
Yes	96 (38%)	30 (25%)	66 (49%)	
No	158 (62%)	90 (75%)	68 (51%)	
**Changed D-Dimer**	**Total (n = 255)**	**Discharge from hospital (n = 121)**	**Death (n = 134)**	**P = 0.600 NS**
Yes	39 (15%)	17 (14%)	22 (16%)	
No	216 (85%)	104 (86%)	112 (84%)	

PaO_2_ = partial pressure of oxygen; PT = prothrombin time; aPTT = activated partial thromboplastin time; NS = not significant; *statistically significant.

The altered levels of PT or aPTT, and anemia, leukocytosis, and thrombocytopenia, were more prevalent in the population that progressed to death. However, the in-hospital mortality was only associated with thrombocytopenia after a logistic regression analysis.

### Treatment characteristics

Although the only treatment recommended by the WHO is the use of systemic corticosteroids in severe or critical cases of COVID-19,^12^ the use of medications in the sample patients was analyzed, as detailed in **eTable 4** in the Supplement (https://drive.google.com/file/d/1WwAiHPfkpZjC1wvaKArI6FpAoEtR-8e9/view).

None of the evaluated medications showed an association with better prognoses in the study population.

The correlation between patients on mechanical ventilation and corticosteroid use was also evaluated. Thus, patients on mechanical ventilation who received corticosteroids had a mortality rate of 72.73% (40/55), whereas for those who did not receive corticosteroids, the rate was 91.89% (102/111) with a P value of 0.006.

Finally, as shown in Table 5, some variables were chosen for the multivariate logistic regression. Therefore, age > 60 years, O_2_ saturation ≤ 94%, use of mechanical ventilation without concomitant corticosteroid therapy, thrombocytopenia, and the use of vasoactive drugs were considered as the risk factors for mortality.

**Table 5.** Multivariate logistic regression of selected variables with odds ratio values, standard error, and significance probability

First group of variables	Total number of patients	OR	OR, 95% CI lower bound	OR, 95% CI upper bound	SE	P
O_2_ saturation	303	3.8	1.2	12.0	0.584	0.022*
Age	316	4.2	1.5	11.5	0.512	0.005*
Thrombocytopenia	292	20.0	4.0	100.5	2.998	< 0.001*
Use of vasoactive drug	316	525.4	122.5	2253.4	0.743	< 0.001*

OR = odds ratio; CI = confidence interval; SE = standard error; O_2_ = oxygen; *statistical significance.

## DISCUSSION

The COVID-19 pandemic is a serious threat to public health owing to the uncertainty it has brought worldwide. The panorama of a new virus with high transmissibility, high heterogeneity, and pathophysiology has not yet been fully elucidated, with cases ranging from an asymptomatic clinical picture to death, achieving a reduction in mortality due to the disease has become a priority in the international scientific community.

Furthermore, reports issued by the WHO show that the mortality rate associated with COVID-19 varies greatly across different countries, indicating that particular geographic features likely play a role in the progression of the disease, and not every study can be extrapolated to individual realities.

In addition, our sample was composed solely of patients from the SUS, the nation’s public healthcare services, on which approximately 70% of the population relies. Therefore, protocols designed to prioritize patients for hospital admission are essential to avoid overcapacity and the consequences of avoidable hospitalization.

Hence, the identification of predictive mortality factors that could aid in the establishment of protocols for the care of the studied population is essential, especially in the management of the most severe cases of the disease spectrum requiring hospitalization, and our study was designed with the aim of filling that gap.

Similar to the findings of other studies that analyzed cohorts of in-hospital patients in the same period as this one, such as the one published by Richardson et al., which analyzed 5,700 patients admitted to hospitals with COVID-19 in the New York City (United States) region,^13^ and the one published by Bellan et al., with 1,697 patients distributed across hospitals in northern Italy,^14^ our study population was characterized by comprising mostly older adult males and patients with comorbidities; being older than 60 years of age can also be considered an independent risk factor for mortality.

Moreover, the mortality rate (51%) in our study was much higher than that reported by Richardson et al.^13^ and Bellan et al.^14^ (21% and 30%, respectively), but similar to those in other Brazilian studies, such as the one by Santos et al.,^15^ a multicenter national study that analyzed 46,285 hospitalized patients due to COVID-19, which reported a 46% mortality rate.

With regard to the characteristics upon admission, an oxygen saturation level ≤ 94%, as measured by pulse oximetry, had an odds ratio of 3.8, i.e, such patients were 3.8-fold more likely to progress to death than those with an oxygen saturation > 94%. The most prevalent symptoms in the sample were dyspnea, coughing, and fever; however, the presence of none may be associated with higher in-hospital mortality.

Although CT scans of the chest accompanied by their respective reports were available for only 37% of patients, they also showed that the greater the degree of pulmonary involvement, the worse the prognosis of the patient. The conclusion remained the same when associated with entry oxygen saturation, i.e., these parameters could be useful for the rapid stratification of cases being admitted to the hospital.

As already explored in other studies,^16^ the hypercoagulable state seems to be associated with higher mortality via pathophysiological mechanisms that are yet to be fully elucidated. This phenomenon can also be seen in our study population, as translated by the association between higher mortality and altered PT and activated thromboplastin time, in addition to thrombocytopenia, which can be considered as a risk factor, with an odds ratio of 20.0, as found by logistic regression.

The use of vasoactive drugs and the need for mechanical ventilation were also highly prevalent in the death group, with the former being considered as a risk factor for such outcomes. However, the concomitant use of corticosteroids in mechanically ventilated patients, as recommended by the WHO,^12^ could result in better prognoses.

This study has a few limitations, primarily due to its observational and retrospective nature; not all the data initially planned for analysis could be retrieved. Therefore, the total sample, comprising 316 patients, could not be analyzed for all the categories initially envisaged. In addition, variables, such as ethnicity and pulmonary involvement as seen on chest CT scans were not only described by a large number of different professionals, but also by dependent examiners. It is also worth noting that the hospital where this study was carried out is a referral facility located in the central region of the city and receives patients transferred from various other less complex minor hospitals/health care system units, which may indicate that the patient’s condition could already be more advanced at the time of the initial care provided by our team and consequently result in worse outcomes and higher mortality.

Our study contributed to determining the phenotype of patients admitted to the hospital with COVID-19 at a higher risk of in-hospital mortality, with the aid of identifying those in need of hospital admission, priority care, case stratification, signs of clinical deterioration, and worse outcomes.

## CONCLUSION

According to our findings, age ≥ 60 years, oxygen saturation ≤ 94% upon admission, use of vasoactive drugs, and thrombocytopenia were the main clinical predictors of in-hospital mortality.

In addition, mechanical ventilation, pulmonary involvement as seen on chest CT scans, altered values of PT, aPTT, leukocytosis, and anemia were more prevalent in the death group; however, they could not be considered as risk factors for mortality after adjustments.
